# Supplementary material for: Is Radiographic Aftercare Obsolete? How Testing Positive for ctDNA Can Be a Precedent for Late Relapse, Even in Low-Risk Hormone-Receptor-Positive Breast Cancer
Source: Int J Mol Sci. 2025 Sep 1;26(17):8498. doi: 10.3390/ijms26178498 (PMC12429740; doi:10.3390/ijms26178498)
Supplement: Supplementary file 1 [file ijms-26-08498-s001.zip › ijms-3685181-supplementary.pdf]

Supplementary Table S1: ctDNA results

Date	eVAF (%)	Score	passing variants	total variants	positive variants	ctDNA – positivity
	estimated variant allele frequency	cumulative statistical evidence from all variants in the panel		of designed panel		
23.04.2018	0.0137	5,97	11	53	4	Yes
18.05.2020	0.0085	3,16	11	53	3	Yes
26.05.2021	0.0044	3,31	11	53	3	Yes
08.07.2022	0.1277	138,17	11	53	10	Yes
18.01.2023	0.1313	185,51	11	53	10	Yes
05.04.2023	ND	-0,04	11	53	0	No

Supplementary Table S2: Imaging results, excluding routine aftercare imaging performed *ex domo* (mamma sonography and mammography once per year)

Date	Imaging Procedure	Result
05/2017	Chest X-ray, upper abdomen sonography and bone scan	No signs of distant metastases
05-06/2021	CT thorax and abdomen; bone scan	No signs of local relapse or distant metastases
12/2021	CT thorax and abdomen; bone scan	No signs of local relapse or distant metastases
06/2022	F-18-FDG PET/CT scan	No signs of local relapse or distant metastases
07/2022	Mammasonography *in domo*, in addition to routine aftercare examinations	No signs of local or contralateral recurrence
01/2023	F-18-FDG PET/CT scan	Locoregional recurrence (cervical lymph nodes)
01/2023	Mammasonography *in domo*, in addition to routine aftercare examinations	Suspicious lymph nodes, biopsy performed
04/2023	CT thorax and abdomen	Treatment response, no new metastases
07/2023	CT thorax and abdomen	Treatment response, no new metastases
09/2023	CT thorax and abdomen	Treatment response, no new metastases
